# Editorial: Resistance to Medical Therapy in Pituitary Tumors

**DOI:** 10.3389/fendo.2022.861230

**Published:** 2022-02-23

**Authors:** Renata S. Auriemma, Manuel D. Gahete, Federico Gatto

**Affiliations:** ^1^ Dipartimento di Medicina Clinica e Chirurgia, Sezione di Endocrinologia, Università Federico II di Napoli, Naples, Italy; ^2^ Maimónides Institute of Biomedical Research of Córdoba (IMIBIC), Córdoba, Spain; ^3^ Department of Cell Biology, Physiology and Immunology, University of Córdoba, Córdoba, Spain; ^4^ Reina Sofía University Hospital, Córdoba, Spain; ^5^ CIBER Pathophysiology of Obesity and Nutrition (CIBERobn), Córdoba, Spain; ^6^ Endocrinology Unit, IRCCS Ospedale Policlinico San Martino, Genova, Italy

**Keywords:** pituitary, resistance, somatostatin receptors, dopamine receptors, tumors, adenomas

Pituitary tumors may exhibit resistance to conventional medical treatments, including somatostatin receptor (SST) ligands (SRLs) and dopamine agonists (DAs) (1, 2). Resistance to medical treatment leads to multi-modal therapies, including surgery and radiotherapy. However, treatment-resistant tumors frequently relapse after radical surgical excision. This is of crucial interest as it occurs in a considerable proportion of patients with pituitary tumors (3). Indeed, resistance to conventional treatments is nowadays considered a predictive factor of the aggressive behavior of the pituitary tumor, being associated with poor prognosis and increased mortality. Predictors of responsiveness to medical treatments are still under investigation, and there is no clear evidence for the best individualized medical therapy in patients with aggressive pituitary lesions.

This Research Topic compiles six original research articles, reviews and minireviews aimed to advance in our knowledge and to shed light into key aspects associated with the resistance to medical therapy in pituitary tumors, addressing clinical features, as well as molecular and genetic mechanisms involved in the resistance to SRL and DA treatment.

In the case of somatotroph tumors, first-generation SRLs (fg-SRLs) still represent the first-line medical therapy for acromegaly (4) due to the well-recognized expression of SSTs, mainly the subtype 2 (SST_2_) and 5 (SST_5_) (5, 6). However, 50% of patients treated with fg-SRLs do not reach biochemical control (namely, normal age-adjusted IGF-1 values or safe GH levels) (3). Therefore, to overcome the current trial-and-error approach commonly used in the medical management of acromegaly, researchers are now focusing on the identification of reliable clinical, radiological and molecular determinants able to predict patient response (7, 8). In this context, Nista et al. evaluated the potential role of various clinical and radiological parameters in predicting the biochemical response to 6-month treatment with fg-SRLs in a cohort of naïve acromegaly patients. The authors demonstrated that younger patients (≤ 40 years-old) are less likely to respond to fg-SRLs, while those subjects with higher age-adjusted IGF-1 values at baseline and harboring a T2-weighted hypointense pituitary adenoma at magnetic resonance imaging (MRI), show a better response to first-line fg-SRL therapy. Of note, the combination of these three parameters was highly predictive of the observed IGF-1 reduction also at multivariable analysis.

In case of (partial) resistance to fg-SRL treatment, current consensus statements and guidelines suggest the use of pasireotide, a multireceptor-targeted SRL, as a valuable treatment option to reach disease control (4, 9). In their extensive review, Puig-Domingo et al. propose a therapeutic algorithm, based on clinical and histopathological patient characteristics, to identify those subjects likely to be poor responders to fg-SRL treatment. In the authors’ view, these patients represent good candidates to a first-line medical approach with pasireotide. In more detail, the presence of a T2-weighted hyperintense MRI signal of the pituitary lesion could already suggest a low chance of achieving disease control by using fg-SRLs. Furthermore, the analysis of tumor specimens (when available), can provide valuable additional information. Indeed, the presence of low SST_2_ expression, sparsely granulated immunohistochemical pattern, as well as low (or mutated) AIP, represent tumor features linked to a poor response to fg-SRL therapy.

As concerns prolactinomas, Yang et al. aimed to address the issue of the variable response to treatment in the peculiar setting of children and adolescents. In this population, the authors found a prevalence of macroadenomas in male patients, together with an overall lower response rate to DA therapy (56%), compared to that reported in adults (>80%) (10). In line with these findings, a high percentage of patients underwent surgical approach (64%), while few patients underwent additional gamma-knife in order to achieve disease control. In conclusion, the authors highlight that the management of prolactinomas in children or adolescence is more complex compared to the general population, thus requiring a multimodal approach.

In addition to clinical features, several studies have tried to identify novel biomarkers able to predict the response to medical treatment in pituitary tumors (11–14). In their mini review, Pivonello et al. provide an overview of the molecular mechanisms associated with the resistance to DAs in pituitary tumors. DAs exhibit an excellent therapeutic response in prolactin-secreting adenomas, as well as in other pituitary tumors with high D2R expression wherein DA therapy does not represent the first-line therapy (but its off-label use is considered). However, DA resistance may occur in a subset of patients. This review highlights that the molecular mechanisms involved in this resistance are not fully elucidated, yet. Anyhow, multiple factors may play a role: i) the reduction of D2R expression, which is regulated by internalization and down-regulation processes orchestrated by β-arrestins and cytoskeleton proteins, such as filamin A (FLNA), ii) changes in the balance of D2R subtypes; iii) regulation of D2R signaling pathway; iv) the increase of angiogenic and fibrotic markers or the abundance of certain miRNAs. In their original research article, Mangili et al. further explored the role of FLNA phosphorylation in pituitary PRL- and ACTH-secreting tumor cells. They demonstrated that FLNA phosphorylation is differently modulated by the activation of cAMP pathway and D2R agonists, which result in increased and decreased phosphorylation, respectively. The Authors found that the inhibition of phospho-FLNA by D2R signaling is required to exert the receptor inhibitory effects on PRL- and ACTH-secreting tumor cells, suggesting that this FLNA modification might represent a new pharmacological target to overcome drug resistance.

In the case of GH-secreting pituitary adenomas, in their mini review Gil et al. highlight the epithelial-to-mesenchymal transition pathway (EMT) as a non-binary process, finely tuned by a complex network of transcription factors. E-cadherin loss (or its nuclear transition) is a key characteristic of EMT, associated with invasiveness and proliferative status in somatotroph tumors. Particularly, a number of studies already demonstrated a link between low E-cadherin levels and a poor response to fg-SRLs in acromegaly patients. Furthermore, the authors point out that other molecules involved in EMT, such as the RAR Related Orphan Receptor C (RORC), might be important regulators of SRL response. Of note, EMT features are emerging as novel therapeutic targets, and the EMT blockade could potentially increase the responsiveness of GH-secreting tumors to SRL therapy.

Altogether, the articles included in this Research Topic add novel evidence about clinical, radiological, genetic and molecular factors involved in the resistance to medical treatment in pituitary tumors. However, these articles suggest that, although some mechanisms can be shared by different pituitary tumors histotypes, there is not a univocal relationship between clinical, radiological or molecular parameters, and pituitary tumors resistance. Therefore, further investigations are strongly needed to improve the current knowledge on this topic.

## Author Contributions

RA, MG, and FG edited this Research Topic and wrote the editorial. All authors contributed to the article and approved the submitted version.

## Conflict of Interest

The authors declare that the research was conducted in the absence of any commercial or financial relationships that could be construed as a potential conflict of interest.

## Publisher’s Note

All claims expressed in this article are solely those of the authors and do not necessarily represent those of their affiliated organizations, or those of the publisher, the editors and the reviewers. Any product that may be evaluated in this article, or claim that may be made by its manufacturer, is not guaranteed or endorsed by the publisher.
